# Latent transition analysis of instrumental activities of daily living in Chinese elderly: based on the 2014–2018 wave of the Chinese Longitudinal Healthy Longevity Survey

**DOI:** 10.1186/s12877-023-04631-5

**Published:** 2024-01-22

**Authors:** Yaqi Wang, Xueying Xu, Jingwen Liu, Qingyun Lv, Hairong Chang, Yuan He, Yue Zhao, Xiaonan Zhang, Xiaoying Zang

**Affiliations:** School of Nursing, Tianjin Medical University, No. 22 Qixiangtai Road, Heping District, 300070 Tianjin, China

**Keywords:** The elderly, Instrumental activities of daily living, Latent transition analysis

## Abstract

**Background:**

The instrumental activities of daily living (IADL) among the elderly have been found to be heterogeneous, with different trajectories. However, the transition of the IADL over time remains unclear. We aimed to explore the transition probabilities and the predictors of IADL among the elderly.

**Methods:**

Longitudinal data from the 2014 (T1) and 2018 (T2) waves of the Chinese Longitudinal Healthy Longevity Survey were extracted. A sample of 2,944 participants aged 65 years or older, with complete responses to the IADL scale, was included. Latent profile analysis (LPA) and latent transition analysis (LTA) were employed to identify latent profiles of IADL and investigate the transition probabilities between profiles from T1 to T2. The predictors of latent profiles and transition probabilities were examined using multinomial regression analysis.

**Results:**

The results of LPA at both T1 and T2 supported a 4-profile model solution. They were labeled as the “*Normal function profile*,” “*Mildly impaired profile*,” “*Moderately impaired profile*,” and “*Highly impaired profile*”. The *Normal function profile* and *Highly impaired profile* were characterized by maintaining stability rather than transitioning over time, with transition probabilities of 0.71 and 0.68, respectively, for maintaining stability. The *Mildly impaired profile* and *Moderately impaired profile* were characterized by a stronger tendency towards transition rather than stability, with transition probabilities of 0.29 and 0.45, respectively, of transitioning to the *Highly impaired profile*. The transition probabilities from the three impaired function profiles to the *Normal function profile* ranged from 0.05 to 0.19. Age, gender, place of residence, and social participation were significant predictors of profile attribution at T1 and transition probabilities over time.

**Conclusions:**

This study employed the LTA to examine the transition probability of IADL among the Chinese elderly. By recognizing the different profiles of IADL and understanding the factors associated with transitions among the elderly, interventions can be tailored to improve their functional independence and successful reintegration into families and society.

**Supplementary Information:**

The online version contains supplementary material available at 10.1186/s12877-023-04631-5.

## Background

The global population of elderly individuals reached approximately 1 billion, and projections suggest that this number will nearly double by 2050 [1]. Developing countries, especially in Asia, are undergoing rapid population aging, resulting in significant changes in population structure and health status [2]. China, in particular, has experienced a notable increase in its elderly population, accounting for 18.70% of the total population in the most recent census conducted in 2021 [3]. This represents a 5.44% increase from the previous census in 2010, positioning China one of the fastest-aging countries globally [3]. The accelerated pace of population aging has created substantial demand on healthcare resources, while also placing strains on social security and economic systems [4]. Population aging affects society in various ways, impacting public policies, healthcare, and social services [5]. Consequently, population aging has emerged as a major global public health concern due to its scale and profound impact on society.

As individuals age, they typically experience a gradual decline in physical function, making them more susceptible to chronic diseases and disabilities that can severely impact their mobility [6]. Instrumental Activities of Daily Living (IADL) are important indicators of an individual’s mobility and functional independence, enabling them to live independently within the community and society [7]. It’s important to distinguish IADL from Basic Activities of Daily Living (BADL), which encompass basic activities like eating, dressing, bathing, and walking [8]. In contrast, IADL involves more complex tasks essential for independent functioning, such as visiting neighbors, shopping, making food, and washing clothes [6]. Research has shown that elderly individuals are more likely to experience impaired IADL compared to BADL [9]. Impaired IADL has been closely associated with various health outcomes [10, 11]. For example, when elderly individuals experience physical limitations that prevent them from performing daily tasks, it results in a loss of independence and significant changes in their lifestyle, leading to a decline in their overall quality of life [10]. Research has also found that impaired IADL in the elderly is linked to frailty, which can result in negative clinical outcomes like disability, readmission, and even mortality [11]. Therefore, by gaining an in-depth understanding of IADL and identifying elderly individuals at high risk for impaired IADL, it becomes possible to provide early evidence for timely identification of health issues and implement tailored interventions to prevent or delay IADL impairment.

Impairments in IADL can stem from a combination of factors [11], highlighting the characteristics that make elderly individuals more susceptible to experiencing such limitations. Research has shown that with increasing age among the elderly, there is an increased risk of severe IADL impairment [12]. Multimorbidity, the presence of multiple chronic diseases, has been identified as a significant predictor of IADL impairment in the elderly [13]. This suggests that having multiple chronic diseases can diminish an individual’s self-care ability and increase their functional dependency. In fact, a study demonstrated that the odds ratio for impaired IADL in individuals with four or more chronic diseases was 4.66 compared to those without any chronic diseases [14]. Residential status also influences IADL, with studies showing that rural residents and those who live alone are more likely to experience IADL impairment [15, 16]. Unhealthy habits such as smoking and alcohol consumption have detrimental effects on IADL [17, 18], contributing to the development of chronic diseases, impaired cognitive function, and weakening physical capabilities. Encouraging elderly individuals to engage in social and leisure activities has been suggested as a way to delay the onset of impaired IADL, regardless of the specific type of activity [19]. Importantly, a study identified three combined profiles of BADL and IADL employing latent profile analysis (LPA) among elderly individuals in China [6]. Another study using a group-based trajectory model demonstrated the dynamic changes of IADL over time, which could be divided into three trajectories [20]. Both studies demonstrated the differences in IADL among elderly individuals based on latent variable modeling, which may be attributed to various demographic characteristics such as age [12], health conditions [13], and their changes over time. These findings indicate that IADL is not a fixed construct and can be influenced by various factors, necessitating further exploration to better understand its transitions and stability over time. The term transition refers to the process by which an individual moves from one state to another over time [21], such as the transition of IADL from normal to impaired. Similarly, maintaining stability refers to the relative constancy of an individual’s state over time [21], for example, maintaining normal IADL over time. By comprehensively understanding these dynamics, healthcare providers can develop targeted interventions to delay negative transitions and promote positive transitions, ultimately improving the overall well-being and functional independence of elderly individuals.

However, there were limitations to the previous studies. First, many of these studies have adopted a variable-centered approach, neglecting to account for potential heterogeneity among participants, with the exception of latent variable modeling methods such as LPA and group-based trajectory models. It is important to consider that the elderly population has diverse needs and varying incidence rates for different IADL items [22, 23]. Similarly, different IADL items may have distinct impacts on the elderly population [22, 23]. For example, research has shown that answering the telephone exhibits the highest impairment rate among IADL items, followed by taking public transportation [22]. Therefore, combined with the research that identified three combined profiles of BADL and IADL, as well as the study that revealed three distinct developmental trajectories of IADL, we can reasonably infer that there may be diversity and heterogeneity in the patterns of IADL impairment in the elderly population. Nonetheless, previous assessments of IADL impairment have primarily relied on cutoff scores [24], which may not accurately capture the varied response patterns exhibited by individuals. This limitation hinders a comprehensive understanding of IADL functioning. Second, most studies have been cross-sectional in nature. Although longitudinal studies have demonstrated the existence of three distinct developmental trajectories for IADL [20], the probability of transitions in the state of IADL over time remains unclear. To address these limitations, latent transition analysis (LTA) may offer a promising solution. The LTA is a person-centered statistical method for analyzing longitudinal data, allowing for the identification and description of the probability of transitions in unobserved latent profiles over time [21], and it has been successfully applied to explore latent transition probabilities of depressive symptoms in the elderly [25].

In the present study, we employed LTA to explore the latent profiles of IADL in the elderly at different time points based on the performance characteristics of the IADL items and investigate the probabilities and predictors of transition in IADL among the elderly over time. Based on the Chinese Longitudinal Healthy Longevity Survey (CLHLS) [26], we propose three hypotheses regarding IADL in the elderly. (1) There is heterogeneity in IADL in the elderly, and it can be divided into different profiles; (2) These latent profiles may maintain stability or transition to other profiles over time; (3) Population characteristics can predict latent profiles and transition probabilities, such as age and social participation [12, 19]. By gaining a clearer understanding of the transition patterns of different IADL profiles using LTA among the elderly over time, further enhancing the exploration and longitudinal understanding of IADL heterogeneity. Healthcare providers can also utilize this evidence to understand the characteristics of different IADL profiles and develop early interventions to reduce the negative transition of IADL impairment among the elderly, ultimately facilitating their return to family and community settings.

## Methods

### Data and study participants

**Fig. 1:**
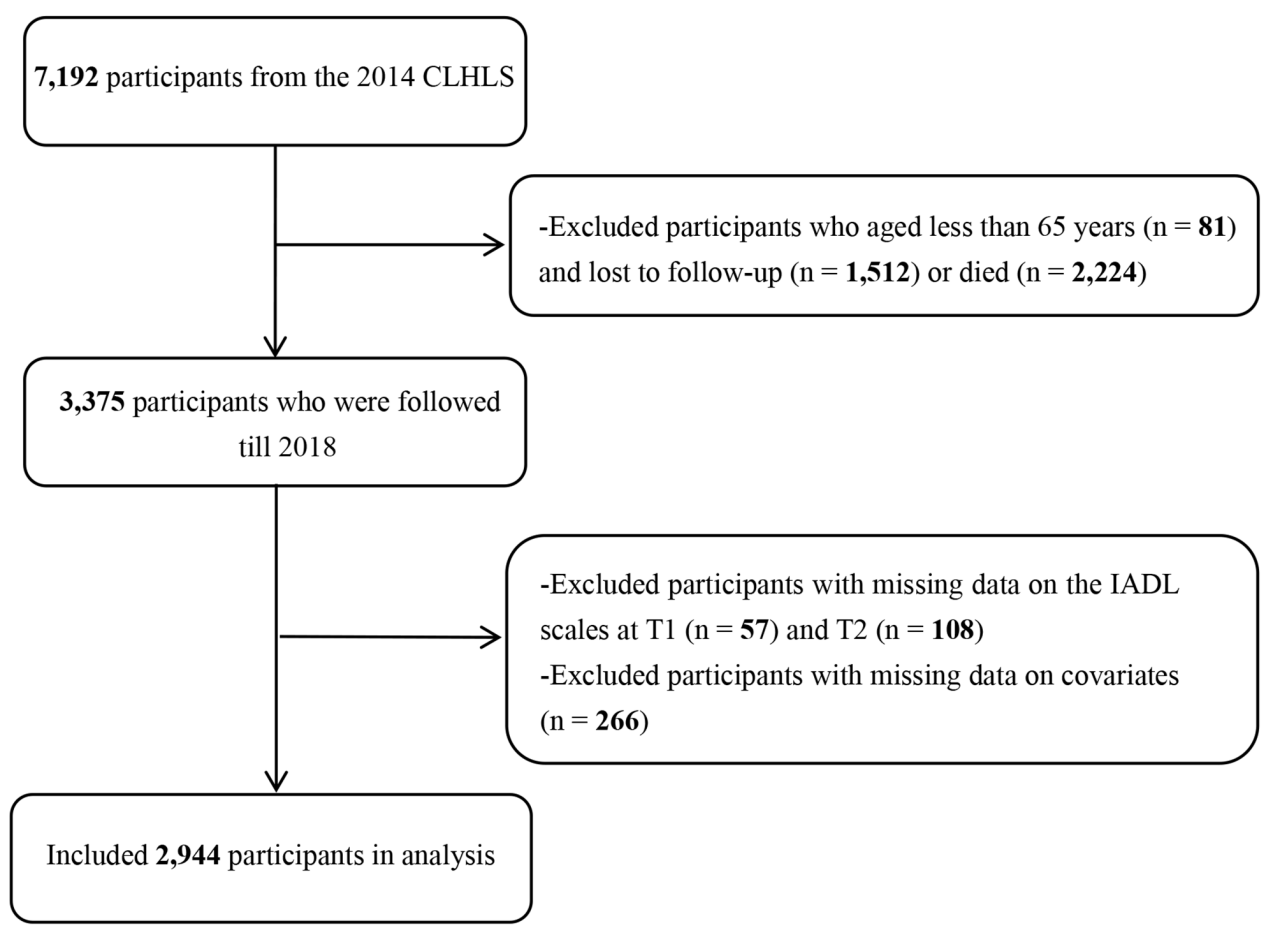
Flow chart of sample selection. Latent profile analysis at T1. (B) Latent profile analysis at T2

The data for this study was obtained from the CLHLS database [26], a comprehensive national survey covering twenty-three provinces in China. The CLHLS collected a wide range of information on family structure, marital status, health status, and socioeconomic factors among the elderly in China. Prior to participation, each participant signed an informed consent form. The most recent data update available at the time of this study was in 2018. We screened the elderly who were 65 or older and participated in both the 2014 (T1) and 2018 (T2) waves with complete responses to the IADL scale. Those with missing covariates were excluded. In the 2014 wave, a total of 7,192 participants were enrolled in the study, with 81 participants below the age of 65. Over the subsequent four years, 3,736 participants were lost to follow-up (n = 1,512) or died (n = 2,224), which were recorded as observed states. Furthermore, 165 participants were missing data for the IADL scales, while an additional 266 participants had incomplete covariate information. Ultimately, our analysis included 2944 (40.9%) participants. The flowchart illustrating the selection of participants for the study is visually depicted in Fig. 1.

### IADL

The IADL scale used in this study is a validated tool for measuring the functional independence of the elderly population [6, 27]. The scale consists of eight items that reflect common tasks and activities experienced by individuals, including visiting neighbors, shopping, making food, washing clothes, walking one kilometer, carrying five kg of weight, crouching and standing three times, and taking public transportation. Each item is scored on a three-point scale, with 1 indicating complete independence, 2 indicating some difficulty, and 3 indicating complete dependence. The total score ranges from 8 to 24, with higher scores indicating more severe impairment in functional independence. The CLHLS sample in 2018 demonstrated a strong internal consistency in IADL, as evidenced by a Cronbach’s alpha coefficient of 0.818 [6]. In the present study, the IADL scale has been shown to possess good internal consistency, with a Cronbach’s alpha of 0.925 for T1 and 0.948 for T2.

### Covariates

Based on the relevant literature [11–19], several demographic characteristics at T1 were selected as covariates, including demographic information, disease information, and social participation [19, 28]. Demographic information included age (measured in years), gender (male/female), place of residence (urban/rural), residential status (living with family/living alone, or in a nursing institution), smoking habits (yes/no), and drinking habits (yes/no). Disease information included the presence or absence of any chronic diseases, with a list of 24 common chronic diseases included in the CLHLS. Participants with one or more of these chronic diseases were deemed to have a chronic disease. Social participation was also included in the analysis, assessed across three dimensions: cognitive activity, physical activity, and social activity [28]. Cognitive activities include “reading newspapers/books,” “playing cards/mah-jong,” “watching TV and listening to the radio”. Physical activities include “doing housework,” “personal outdoor activities,” “garden work,” “raising domestic animals/pets,” and “exercising or not at present”. Social activities include “taking part in some social activities” and “traveling beyond home city/county in the past two years”. The total score ranges from 0 to 34, with higher scores indicating greater engagement in social activities.

### Statistical analysis

The data analysis was performed in the following four steps. In step 1, based on clinical practice and relevant literature [6], in order to obtain concise and interpretable models, two to five latent profile models based on each of the eight items of the IADL scale from T1 to T2 were explored separately using latent profile analysis (LPA). The best model was chosen based on a comprehensive consideration of the following fit indices. Lower values of the Akaike information criterion (AIC) [29], the Bayesian information criterion (BIC) [30], and the Sample-adjusted Bayesian Information Criterion (aBIC) indicate a better-fitting model [31]. Lower values of the Vuong-Lo-Mendell-Rubin (VLMR) and Bootstrapped Likelihood Ratio Test (BLRT) indicate that the K-profile model is better than the K-1 profile model [32]. Greater entropy indicates higher classification accuracy, with a value greater than 0.8 indicates that the classification accuracy is greater than 90% [33]. To ensure robust exploration of predictors and representative profile samples, the minimum group size was set at 5% of the total sample. Once the optimal profile model was identified, profiles were named based on the characteristics of item means within each profile. Multinomial logistic regression was then used to explore the predictors of the profiles at T1, with the profile showing the least functional impairments serving as the reference group. In step 2, LTA was employed to examine the transition probabilities between profiles from T1 to T2, without considering covariates. In step 3, odds ratios (OR) for a profile to transition to other profiles with covariates from T1 to T2 were explored with reference to remaining in the original profile. An OR smaller than 1 indicates that, under the influence of a particular covariate, the elderly were less likely to transition to other profiles compared to remaining in their original profile. Conversely, an OR greater than 1 suggests a higher likelihood of transitioning to other profiles. In step 4, we conducted the sensitivity analyses. These included interpolating IADL information for elderly participants who completed follow-up but had incomplete responses. The three aforementioned steps were repeated to assess the stability of the main results. All statistical analyses were conducted using Mplus (version 8.0, Muthén, 2007) and SPSS (version 25.0, Armonk, 2017).

## Results

### Descriptive statistics

Of the 2944 elderly participants, the mean age was 81.9 ± 8.6 years old, with 1460 (47.7%) being male. Among them, 1265 (43%) resided in urban areas, 2265 (76.9%) lived with their families, 533 (18.1%) were smokers, 519 (17.6%) were alcohol drinkers, and 609 (20.6%) had chronic diseases. The mean social participation score was 12.0 ± 6.0. The mean IADL score was 10.7 ± 4.3 for T1 and 13.2 ± 5.8 for T2. Compared to the elderly individuals included in the analysis, those excluded are more likely to be female, older in age, residing in urban areas, living with family members, non-smokers, non-drinkers, having chronic diseases, and experiencing lower levels of social participation and more severe impairment in IADL (Table S1).

### LPA model selection

The fit indices of all profile models for the IADL at T1 and T2 are shown in Table 1, with a consistent pattern observed across both T1 and T2. As the number of profiles increased, the values of AIC, BIC, and aBIC decreased, indicating improved model fit. The entropy values for all models were greater than 0.8, indicative of high classification accuracy (greater than 90%). However, the P-values of VLMR for the 5-profile model were greater than 0.05 at both T1 and T2, indicating that the 5-profile model was not significantly better than the 4-profile model. Additionally, the minimum group sizes in the 5-profile model accounted for less than 5%, potentially leading to uneven distribution among profiles and affecting predictor exploration. Thus, after comprehensive consideration of the characteristic fit indices, the 4-profile model was selected as the best-fit model for both T1 and T2. The model fitting results were consistent across sensitivity analysis (Table S2).

**Table 1:** The fit indices of the all profile models at T1 and T2

Time	Profile	AIC	BIC	aBIC	Entropy	VLMR (*P*)	BLRT (*P*)	Minimum sizes
T1								
	2	30,801.853	30,951.541	30,872.107	0.990	< 0.001	< 0.001	0.14
	3	25,592.203	25,795.779	25,687.748	0.976	< 0.001	< 0.001	0.07
	4	22,080.491	22,337.955	22,201.328	0.976	< 0.001	< 0.001	0.07
	5	19,930.738	20,242.089	20,076.865	0.985	0.060	0.034	0.03
T2								
	2	39,628.602	39,778.290	39,698.856	0.984	0.018	< 0.001	0.28
	3	33,882.494	34,086.069	33,978.039	0.970	0.003	< 0.001	0.19
	4	30,434.613	30,692.077	30,555.450	0.978	< 0.001	< 0.001	0.14
	5	28,676.970	28,988.321	28,823.097	0.977	0.051	0.003	0.04

Abbreviations: AIC, the Akaike information criterion; BIC, the Bayesian information criterion (BIC); aBIC, the Sample-adjusted Bayesian Information Criterion (aBIC); VLMR, the Vuong-Lo-Mendell-Rubin; BLRT, Bootstrapped Likelihood Ratio Test (BLRT).

### Profile characteristics

**Fig. 2:**
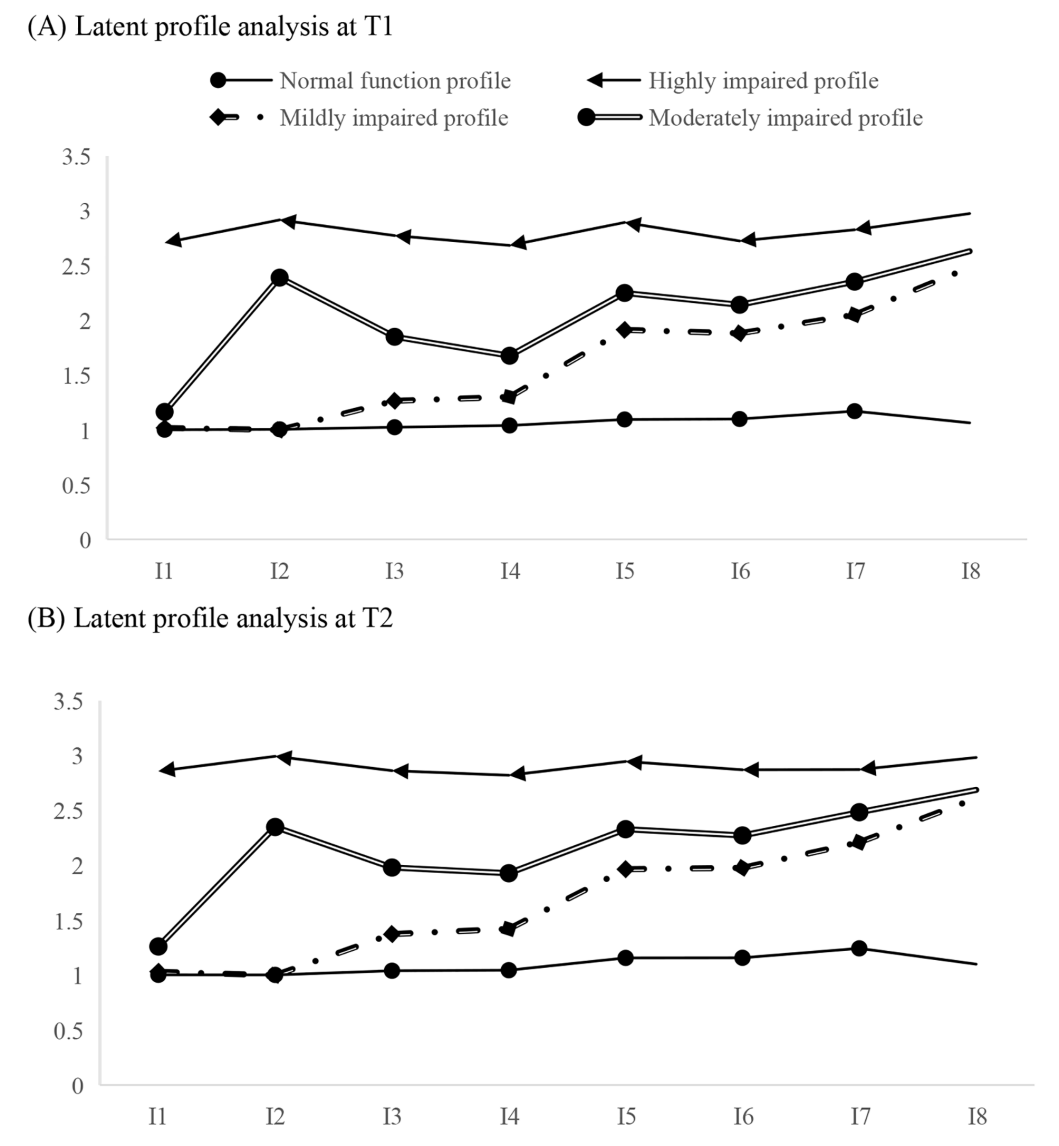
The IADL item means for each profile in the 4-profile model at T1 and T2. I1, visiting neighbors; I2, shopping; I3, making food; I4, washing clothes; I5, walking one kilometer; I6, carrying five kg of weight; I7, crouching and standing three times; I8, taking public transportation

**Fig. 3:**
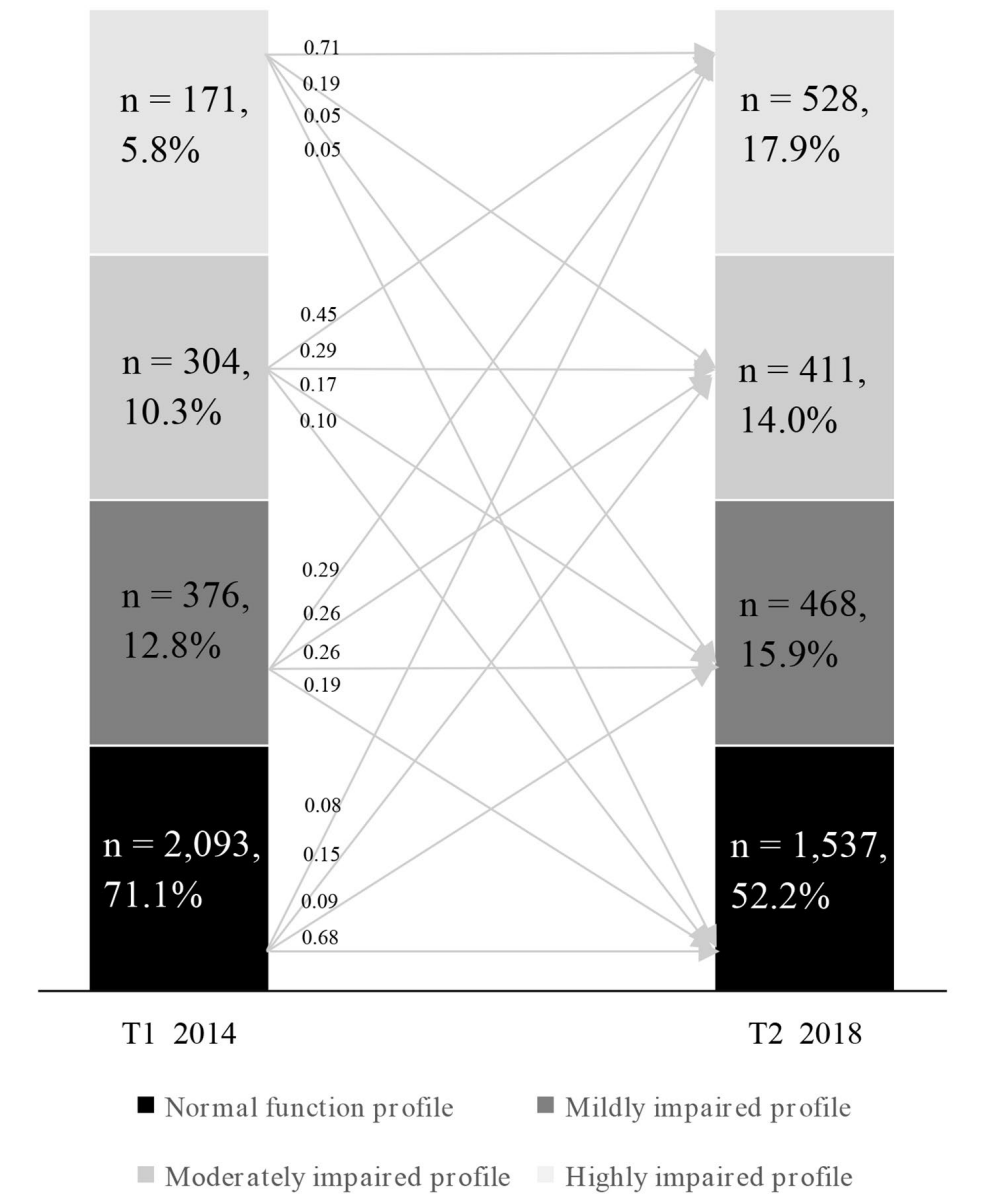
The percentage in each of the four profiles and transition probabilities from T1 to T2

The estimated profiles of the 4-profile model are plotted in Fig. 2. The item means of the curve at the bottom closely approximated “1”, indicating normal functioning of IADL. Hence, it was named the “*Normal function profile*”. The item means of the curve at the top approached “3”, implying severe impairment in IADL. Therefore, it was named the “*Highly impaired profile*”. The item means of two curves in the middle scored between “1” and “3”, suggesting varying degrees of IADL impairments. Based on the extent of impairments, they were named the “*Mildly impaired profile*” and the “*Moderately impaired profile*”, respectively. The proportions of the elderly in each profile at both T1 and T2 are shown in Fig. 3. The results of the sensitivity analysis were consistent (Fig. S1 and S2).

### Profile predictors

To explore the predictors of different IADL profiles at T1, a multinomial logistic regression model was constructed, including all covariates, with the *Normal function profile* as the reference group. The results are shown in Table 2. Increasing age was associated with a higher likelihood of developing IADL impairment (OR ranged from 1.08 to 1.12, all *P* < 0.01). As social participation increased, the likelihood of IADL impairment decreased (all OR < 1.00, *P* < 0.01). Elderly individuals residing in rural areas were more likely to exhibit highly impaired IADL (OR = 3.00, *P* < 0.01). Those without co-morbidities had a lower probability of experiencing IADL impairment (OR ranged from 0.41 to 0.62, *P* < 0.01). Whereas smoking habits, drinking habits, and residential status had no significant effect on IADL impairment. Similar results were obtained in the sensitivity analysis (Table S3).

**Table 2:** Odds Ratio reflecting effects of predictors of different profiles at baseline

Predictor	Mildly impaired	Moderately impaired	Highly impaired
Age	1.08^**^	1.11^**^	1.12^**^
Cognitive activity	0.89^**^	0.85^**^	0.80^**^
Physical activity	0.92^**^	0.83^**^	0.54^**^
Social activity	0.75^**^	0.82	0.69
Place of residence (ref = rural)	1.13	1.44	3.00^**^
Gender (ref = female)	0.45^**^	0.39^**^	0.23^**^
Residential status (ref = live alone or in a nursing institution)	0.80	1.04	1.25
Smoking habits (ref = no)	1.06	0.76	0.50
Drinking habits (ref = no)	0.77	0.73	0.92
Chronic disease (ref = yes)	0.62^**^	0.61^**^	0.41^**^

Ref = Normal function profile

^*^P < 0.05; ^**^P < 0.01

### Profile stability and transition probability

The transition probabilities across profiles, without covariates, from T1 to T2 are plotted in Fig. 2. The results indicated that both the *Normal function profile* and *Highly impaired profile* were largely stable, with probabilities of 0.68 and 0.71, respectively. The transition probabilities between these two profiles were very low, with values of 0.05 and 0.08. Transitions were more significant in the *Mildly impaired profile* and *Moderately impaired profile*, but they tended to be negative rather than positive. The transition probabilities from the *Mildly impaired profile* to the *Highly impaired profile* and the *Moderately impaired profile* were 0.29 and 0.26, respectively. The *Moderately impaired profile* had the greatest transition probability of 0.45 to the *Highly impaired profile*. The transition probability from the three IADL-impaired profiles to the *Normal function profile* ranged from 0.05 to 0.19 Additional details on the transition probabilities are shown in Fig. 2. The sensitivity analysis showed similar transition probabilities (Table S2).

### Covariate effects

To explore the effect of covariates on the transition probabilities between profiles, ORs were calculated using staying in the original profile from T1 to T2 as the reference group. The results are shown in Table 3. As age increased, the probability of transitioning to a worse IADL profile intensified. From T1 to T2, the transition probabilities from the *Normal function profile* to the three impaired profiles increased (OR ranged from 1.09 to 1.14, all *P* < 0.01). For those in the *Mildly impaired profile*, the transition probabilities to the *Moderately impaired profile* and *Highly impaired profile* also increased (OR = 1.08 and 1.11, all *P* < 0.01). The transition probabilities from the *Moderately impaired profile* to the *Normal function profile* and *Mildly impaired profile* decreased (OR = 0.90 and 0.93, *P* < 0.01, < 0.05, respectively). Higher levels of social participation were found to facilitate maintaining the original profile or transitioning to a better profile. Increased cognitive decreased the probability of transitioning from *Normal function profile* to the *Mildly impaired profile* (OR = 0.93, *P* < 0.01). However, for individuals in the *Highly impaired profile*, despite increased cognitive activity, they were more likely to remain in that profile rather than transitioning to the *Moderately impaired profile* (OR = 0.76, *P* < 0.05). Enhanced physical activity in the *Normal function profile* promoted remaining in the original profile instead of transitioning to the *Highly impaired profile* (OR = 0.95, *P* < 0.05). In contrast, for individuals in the *Highly Impaired profile*, increased physical activity facilitated transitioning to the *Mildly impaired profile*, *Moderately impaired profile*, and *Normal function profile* (OR = 1.35, 1.28, and 1.57, all *P* < 0.05). With increasing social activity, the elderly in the *Normal function profile* were more likely to remain in the original profile rather than transitioning to the *Mildly impaired profile* (OR = 0.85, *P* < 0.05). More information can be found in Table 3. The sensitivity analysis showed similar results (Table S4).

**Table 3:** Odds Ratio for covariates predicting transitions among profiles

		T2 latent profile			
Predictor	T1 latent profile	Normal function	Mildly impaired	Moderately impaired	Highly impaired
Age	Normal function	ref	1.09^**^	1.14^**^	1.13^**^
	Mildly impaired	0.98	ref	1.08^**^	1.11^**^
	Moderately impaired	0.90^**^	0.93^**^	ref	1.03
	Highly impaired	0.93	0.99	0.99	ref
Cognitive activity	Normal function	ref	0.93^**^	0.95	0.95
	Mildly impaired	1.01	ref	0.97	1.12
	Moderately impaired	1.08	1.06	ref	0.96
	Highly impaired	0.95	0.75	0.76^*^	ref
Physical activity	Normal function	ref	1.00	1.03	0.95^**^
	Mildly impaired	1.06	ref	0.99	0.96
	Moderately impaired	1.11	0.97	ref	0.92^*^
	Highly impaired	1.57^**^	1.35^*^	1.28^*^	ref
Social activity	Normal function	ref	0.85^*^	0.85	0.94
	Mildly impaired	0.96	ref	0.86	0.79
	Moderately impaired	1.45	1.50	ref	1.31
	Highly impaired	< 0.01^a^	2.20	1.39	ref
Place of residence(ref = rural)	Normal function	ref	0.55	0.84	0.86
	Mildly impaired	0.84	ref	1.06	2.34
	Moderately impaired	2.27	0.95	ref	2.07
	Highly impaired	0.97	3.86	1.32	ref
Gender(ref = female)	Normal function	ref	0.66^**^	0.64^*^	0.65^*^
	Mildly impaired	0.96	ref	0.70	0.76
	Moderately impaired	1.21	0.74	ref	0.89
	Highly impaired	3.67	2.78	1.98	ref
Residential status(ref = live alone or in a nursing institution)	Normal function	ref	0.83	0.84	1.56
	Mildly impaired	1.07	ref	1.39	1.71
	Moderately impaired	0.61	0.53	ref	0.88
	Highly impaired	0.36	0.55	0.39	ref
Smoking habits(ref = no)	Normal function	ref	0.90	0.79	0.86
	Mildly impaired	1.05	ref	0.75	0.48
	Moderately impaired	1.29	2.44	ref	1.30
	Highly impaired	0.57	< 0.01^a^	5.94	ref
Drinking habits(ref = no)	Normal function	ref	1.33	0.91	0.96
	Mildly impaired	1.61	ref	1.24	1.11
	Moderately impaired	1.44	0.50	ref	0.41
	Highly impaired	1.50	< 0.01^a^	< 0.01^a^	ref
Chronic disease(ref = yes)	Normal function	ref	1.01	0.75	0.62^*^
	Mildly impaired	0.50	ref	0.70	0.70
	Moderately impaired	1.07	1.54	ref	0.75
	Highly impaired	0.50	3.12	1.16	ref

^*^P < 0.05; ^**^P < 0.01

^a^ Odds Ratio< 0.01

## Discussion

In this study, data from the 2014–2018 wave of the CLHLS was analyzed to explore the latent profiles of IADL among the Chinese elderly. The analysis identified the presence of four distinct profiles: *Normal function*, *Mildly impaired*, *Moderately impaired*, and *Highly impaired profile*s. The profiles exhibited different transition characteristics over the four-year period. The study found that the *Normal function profile* and the *Highly impaired profile* demonstrated stability, with low transition probabilities observed. In contrast, the *Mildly impaired profile* and *Moderately impaired profile* displayed significant transitions, primarily in a negative direction. Elderly individuals in these profiles were more likely to transition to the *Highly impaired profile*, with the *Moderately Impaired profile* exhibiting a particularly high probability (0.45), indicating a deterioration in IADL. Additionally, the transition probabilities from the three profiles to the *Normal function profile* were found to be low, suggesting that without intervention, reversing IADL impairment is less likely. The study’s conclusions emphasized the importance of targeted interventions and support for elderly individuals with IADL impairments. Age, gender, social participation, and chronic diseases were identified as important predictors of both the original profile classification and the subsequent transitions between profiles. The results of the sensitivity analysis are consistent with the main analysis results, indicating the stability of the research conclusions.

There was a notable heterogeneity observed in the profiles of IADL among the elderly population. Previous studies have suggested that IADL impairment is a gradual process of change, indicating the existence of different profiles of IADL impairment in a sufficiently large and heterogeneous sample [20, 34], which is consistent with our conclusions. However, in another related study, three combined profiles of BADL and IADL were identified among the elderly [6], which was inconsistent with the result of four IADL profiles in this study. One possible explanation for this difference could be that the inclusion of BADL may impact the exploration of IADL profiles. One of the strengths of this study is that the identification of these four profiles holds greater clinical reference value, which can provide health care providers with a more detailed assessment of the extent of IADL impairment in the elderly. The study’s results suggest a gradual deterioration of IADL over time, with a decrease in the proportion of elderly individuals in the *Highly impaired profile* (from 71.1 to 52.2%) and an increase in the proportion of individuals in the other three impaired IADL profiles. This underscores the importance of implementing multi-time point monitoring of IADL in the elderly and intervening to prevent deterioration.

This study also provided valuable insights into the predictions of different profiles. Age emerged as a risk factor for impaired function, consistent with the findings of other study [35]. IADL impairment is primarily affected by aging and declining physical function, so the elderly have more severe IADL impairment [36]. While high social participation was associated with a lower likelihood of impaired IADL, the study revealed that this association was only significant for mild IADL impairment and not for moderate or severe impairment. Research suggests that elderly individuals with IADL impairment may experience social isolation [37], which may limit their social activities or counteract the protective effects of social activities. Meanwhile, the other study found that IADL was a predictor of social participation trajectories, with poorer IADL predicting poorer social participation [28]. Thus, there may be a bidirectional relationship between impaired IADL and reduced social participation. A meta-analysis has suggested that muscle strength was a prerequisite for performing IADL [38], explaining the higher likelihood of impaired IADL among elderly individuals in urban areas, where individuals may have experienced reduced physical activity compared to their rural counterparts [39]. Additionally, gender differences in IADL impairment were noted, with females exhibiting a higher probability of impairment, potentially influenced by societal roles and domestic responsibilities [40]. Additionally, this study found that the elderly without chronic diseases were less likely to have impaired IADL than those with chronic diseases. This can be attributed to the negative impact of chronic diseases on physical function, which restricts the individual’s ability to engage in daily activities [41, 42].

The different profiles of the elderly showed different major transition characteristics. The primary characteristic of both the *Normal function profile* and the *Highly impaired profile* was maintaining stability. Despite some elderly individuals transitioning to other profiles over the course of four years, the *Normal function profile* remained predominant due to its larger baseline representation (71.1%). These findings suggest that a longer observation period may be necessary to observe more significant transitions between profiles. The highly impaired IADL represents advanced disease progression or aging, leading to an increased number of individuals in this profile. Transitions were more commonly observed in the *Mildly and Moderately impaired profile*s, with predominantly negative changes, suggesting a chronic exacerbation of IADL impairment [43]. However, a subset of the elderly did experience positive transitions. It is noteworthy that the transition probabilities were higher in the *Mildly and Moderately impaired profiles* compared to the *Highly impaired profile*, indicating a decrease in the probability of natural improvement as the level of impairment increases. Another study has also shown that IADL impairment tends to worsen in the elderly without interventions imposed [20]. Therefore, early identification of the elderly with IADL impairment and targeted interventions are important. The following findings may help identify factors associated with transition and propose targeted interventions. With aging, IADL declined. The influence of aging on physical function is inevitable, thus increasing the possibility of impaired or deteriorating IADL in the elderly [44]. Enhanced physical and social activities in social participation have been shown to help maintain stability or facilitate positive transitions. However, interestingly, increasing cognitive activities in social participation did not lead to positive transitions to the *Moderately impaired profile* among the elderly in the *Highly impaired profile*. Possible reasons for this finding could be the presence of stronger opposing factors, such as cognitive impairment [45]. This suggests that when severe physical limitations are present, increasing cognitive activities may have limited impact on reversing impairment. Therefore, although social participation may have a protective effect on IADL impairment [28], the effects may differ depending on the type of social participation. It is possible that interventions focusing on physical activity and social interactions may yield better results, but further research is needed to confirm this. Men were found to be more likely than women to maintain normal function without transitioning to IADL impairment, potentially due to greater muscle strength [38]. Therefore, future interventions should prioritize targeting women to support their maintenance of IADL function. Furthermore, the elderly without chronic diseases were also more likely to remain in the *Normal function profile*. This suggests that managing chronic diseases is crucial for preventing or minimizing IADL impairment among the elderly [41].

This study is significant as it provides valuable insights into the latent profile characteristics, transition probabilities, and predictors of IADL in the elderly population in China. We employed a novel approach by categorizing individuals into distinct profiles based on similar response patterns, allowing for a more comprehensive understanding of the heterogeneity within the population. Additionally, the results of LTA modeled the transition probabilities between the four IADL profiles, which contributes to a deeper understanding of IADL dynamics. It is worth noting that the IADL scale itself does not provide more rational cutoff values for reference, which means that traditional methods relying on such cutoffs may not fully capture this complexity. Early identification of the elderly with impaired IADL and the implementation of targeted interventions aimed at preventing deterioration or negative transition in IADL may effectively improve their physical function in the context of the growing aging problem, ultimately facilitating their return to families and society.

There were several limitations associated with this study. First, it is important to acknowledge the high drop rate observed due to the unfortunate reality that individuals with severe IADL impairments may have passed away during the four-year study period. Additionally, the uneven distribution of included and excluded samples may introduce certain biases. Nevertheless, sensitivity analyses conducted specifically on the surviving elderly individuals showed consistent or similar results with the main results, indicating the stability of the research conclusions. Second, it is worth noting that the reliance on self-assessment within the IADL scale may introduce an inherent self-reporting bias into the data. Third, the observation period was limited to four years based on the available database. Consequently, conducting additional assessments at multiple time points would be beneficial for obtaining a more comprehensive understanding of the dynamic characteristics of IADL transitions among the elderly population. Fourthly, as this study exclusively focused on the elderly in China, caution should be exercised when generalizing these findings to elderly populations in other countries.

## Conclusion

In conclusion, this study confirmed the existence of distinct IADL profiles and transition probabilities among the elderly population. The results highlighted the necessity for supportive interventions aimed at fostering family and social reintegration in the context of rapid population aging. Future intervention studies should pay particular attention to vulnerable populations such as women and those with low social participation, as they may be at a higher risk of negative transitions in IADL over time.

### Electronic supplementary material

Below is the link to the electronic supplementary material.

Supplementary Material 1

## Data Availability

Center for Healthy Aging and Development Research, Peking University, 2020, “Chinese Survey of Factors Influencing Healthy Longevity in Older Adults (CLHLS)-Tracking Data (1998–2018)”, 10.18170/DVN/WBO7LK.

## Abbreviations

BADL: Basic Activities of Daily Living
IADL: Instrumental Activities of Daily Living
CLHLS: The Chinese Longitudinal Healthy Longevity Survey

## References

[1] WHO. Ageing and Health. Available online: https://www.who.int/news-room/fact-sheets/detail/ageing-and-health.

[2] Balachandran A, de Beer J, James KS, van Wissen L, Janssen F (2020). Comparison of Population Aging in Europe and Asia using a time-consistent and comparative aging measure. J Aging Health.

[3] Zhai W. 2021. Interpretation of the communique of the Seventh National Population Census. Retrieved from. https://www.pkulaw.com/en_law/a9624a5c331765f8bdfb.html.

[4] Man W, Wang S, Yang H (2021). Exploring the spatial-temporal distribution and evolution of population aging and social-economic indicators in China. BMC Public Health.

[5] Martin LG (1991). Population aging policies in East Asia and the United States. Science.

[6] Zhang Y, Xiong Y, Yu Q, Shen S, Chen L, Lei X (2021). The activity of daily living (ADL) subgroups and health impairment among Chinese elderly: a latent profile analysis. BMC Geriatr.

[7] Fuchs J, Gaertner B, Prütz F (2022). Limitations in activities of daily living and support needs - analysis of GEDA 2019/2020-EHIS. J Health Monit.

[8] Albert SM, Bear-Lehman J, Burkhardt A (2009). Lifestyle-adjusted function: variation beyond BADL and IADL competencies. Gerontologist.

[9] Gong B, Wu C (2021). The mediating and moderating effects of depression on the relationship between cognitive function and difficulty in activities of daily living among postmenopausal women. Menopause.

[10] Beltz S, Gloystein S, Litschko T, Laag S, van den Berg N (2022). Multivariate analysis of Independent determinants of ADL/IADL and quality of life in the elderly. BMC Geriatr.

[11] Kojima G, Quick, Simple FRAIL (2018). Scale Predicts Incident activities of Daily Living (ADL) and instrumental ADL (IADL) disabilities: a systematic review and Meta-analysis. J Am Med Dir Assoc.

[12] Chauhan S, Kumar S, Bharti R, Patel R (2022). Prevalence and determinants of activity of daily living and instrumental activity of daily living among elderly in India. BMC Geriatr.

[13] Soh CH, Hassan SWU, Sacre J, Lim WK, Maier AB (2021). Do morbidity measures predict the decline of activities of daily living and instrumental activities of daily living amongst older inpatients? A systematic review. Int J Clin Pract.

[14] Qiao Y, Liu S, Li G (2021). Longitudinal Follow-Up studies on the Bidirectional Association between ADL/IADL disability and multimorbidity: results from two national sample cohorts of Middle-aged and Elderly adults. Gerontology.

[15] Qian J, Ren X (2016). Association between comorbid conditions and BADL/IADL disability in Hypertension patients over age 45: based on the China health and retirement longitudinal study (CHARLS). Med (Baltim).

[16] Henning-Smith C, Shippee T, Capistrant B (2018). Later-Life disability in Environmental Context: why living arrangements Matter. Gerontologist.

[17] Liu H, Jiao J, Zhu C (2020). Potential associated factors of functional disability in Chinese older inpatients: a multicenter cross-sectional study. BMC Geriatr.

[18] Li G, Li K (2022). Turning point of Cognitive decline for Chinese older adults from a longitudinal analysis: protective factors and risk factors. Healthc (Basel).

[19] Ukawa S, Tamakoshi A, Tani Y (2022). Leisure activities and instrumental activities of daily living: a 3-year cohort study from the Japan gerontological evaluation study. Geriatr Gerontol Int.

[20] Yang J, Zhang Y, Shen S (2023). Instrumental activities of daily living trajectories and risk of mild cognitive impairment among Chinese older adults: results of the Chinese longitudinal healthy longevity survey, 2002–2018. Front Public Health.

[21] Muthén B, Asparouhov T (2022). Latent transition analysis with random intercepts (RI-LTA). Psychol Methods.

[22] Zhang H, Wang ZH, Wang LM, Qi SG, Li ZX (2019). Zhonghua Liu Xing Bing Xue Za Zhi.

[23] Chen S, Zheng J, Chen C (2018). Unmet needs of activities of daily living among a community-based sample of disabled elderly people in Eastern China: a cross-sectional study. BMC Geriatr.

[24] Zhao L, Wang J, Deng H, Chen J, Ding D (2022). Depressive symptoms and ADL/IADL disabilities among older adults from low-income families in Dalian, Liaoning. Clin Interv Aging.

[25] Ni Y, Tein JY, Zhang M, Yang Y, Wu G (2017). Changes in depression among older adults in China: a latent transition analysis. J Affect Disord.

[26] Center for Healthy Aging and Development Research, University P. 2020, Chinese survey of factors influencing healthy longevity in older adults (CLHLS)-Tracking Data (1998–2018), 10.18170/DVN/WBO7LK.

[27] Wei K, Liu Y, Yang J (2022). Living arrangement modifies the associations of loneliness with adverse health outcomes in older adults: evidence from the CLHLS. BMC Geriatr.

[28] Zhang C, Zhao Y, Chen X (2023). Trajectories of Social Participation and its predictors in older adults: based on the CLHLS cohorts from 2002 to 2018. Int J Environ Res Public Health.

[29] Vrieze SI (2012). Model selection and psychological theory: a discussion of the differences between the Akaike information criterion (AIC) and the bayesian information criterion (BIC). Psychol Methods.

[30] Lorah J, Womack A (2019). Value of sample size for computation of the bayesian information criterion (BIC) in multilevel modeling. Behav Res Methods.

[31] Mulder J, Gu X (2022). Bayesian testing of scientific expectations under Multivariate Normal Linear models. Multivar Behav Res.

[32] Harrell PT, Mancha BE, Martins SS (2014). Cognitive performance profiles by latent classes of drug use. Am J Addict.

[33] Larose C, Harel O, Kordas K, Dey DK (2016). Latent class analysis of Incomplete Data via an Entropy-based Criterion. Stat Methodol.

[34] Wassenius C, Claesson L, Blomstrand C, Jood K, Carlsson G (2022). Integrating consequences of Stroke into everyday life - experiences from a long-term perspective. Scand J Occup Ther.

[35] Tabira T, Hotta M, Murata M (2020). Age-related changes in Instrumental and Basic activities of Daily living impairment in older adults with very mild Alzheimer’s Disease. Dement Geriatr Cogn Dis Extra.

[36] McGrath R, Vincent BM, Hackney KJ (2020). Weakness and cognitive impairment are independently and jointly associated with functional decline in aging americans. Aging Clin Exp Res.

[37] Wong EL, Qiu H, Cheung AW, Leung HH, Chen FY, Yeoh EK (2023). Association of social isolation with health status among community-dwelling Chinese older adults living with homecare services: a cross-sectional survey in Hong Kong. Front Public Health.

[38] Wang DXM, Yao J, Zirek Y, Reijnierse EM, Maier AB (2020). Muscle mass, strength, and physical performance predicting activities of daily living: a meta-analysis. J Cachexia Sarcopenia Muscle.

[39] Archer J (1996). Sex differences in social behavior. Are the social role and evolutionary explanations compatible?. Am Psychol.

[40] Eagly AH, Wood W (1991). Explaining sex differences in social behavior: a meta-analytic perspective. Pers Soc Psychol Bull.

[41] Wang Z, Peng W, Li M (2021). Association between multimorbidity patterns and disability among older people covered by long-term care insurance in Shanghai, China. BMC Public Health.

[42] Chou CY, Chiu CJ, Chang CM (2021). Disease-related disability burden: a comparison of seven chronic conditions in middle-aged and older adults. BMC Geriatr.

[43] Fieo R, Zahodne L, Tang MX, Manly JJ, Cohen R, Stern Y (2018). The historical progression from ADL scrutiny to IADL to Advanced ADL: assessing functional status in the Earliest stages of Dementia. J Gerontol A Biol Sci Med Sci.

[44] McGarrigle CA, Ward M, Kenny RA (2022). Negative aging perceptions and cognitive and functional decline: are you as old as you feel?. J Am Geriatr Soc.

[45] Passler JS, Kennedy RE, Clay OJ (2020). The relationship of longitudinal cognitive change to self-reported IADL in a general population. Neuropsychol Dev Cogn B Aging Neuropsychol Cogn.

