# Supplementary material for: Latent transition analysis of instrumental activities of daily living in Chinese elderly: based on the 2014–2018 wave of the Chinese Longitudinal Healthy Longevity Survey
Source: BMC Geriatr. 2024 Jan 22;24:83. doi: 10.1186/s12877-023-04631-5 (PMC10804623; doi:10.1186/s12877-023-04631-5)
Supplement: Supplementary file 1 — Supplementary Material 1 [file 12877_2023_4631_MOESM1_ESM.docx]

**Latent transition analysis of instrumental activities of daily living in Chinese elderly: based on the 2014–2018 wave of the Chinese Longitudinal Healthy Longevity Survey**

**SUPPLEMENTAL FILES**

- Supplemental Table 1. Comparison of baseline characteristics between participants included (n = 2,944) and excluded (n = 4,248).
- Supplemental Table 2. The fit indices of the all profile models at T1 and T2 based on sensitivity analysis (n = 3,375).
- Supplemental Table 3. Odds Ratio reflecting effects of predictors of different profiles at baseline based on sensitivity analysis (n = 3,375).
- Supplemental Table 4. Odds Ratio for covariates predicting transitions among profiles based on sensitivity analysis (n = 3,375).
- Supplemental Fig 1. The IADL item means for each profile in the 4-profile model at T1 and T2 based on sensitivity analysis (n = 3,375).
- Supplemental Fig 2. The percentage in each of the four profiles and transition probabilities from T1 to T2 based on sensitivity analysis (n = 3,375).

Supplemental Table 1. Comparison of baseline characteristics between participants included (n = 2,944) and excluded (n = 4,248).

| Characteristic | Included  (N = 2,944) | Excluded  (N = 4,248) | *P* Value |
| --- | --- | --- | --- |
| Gender, n (%) |  |  |  |
| Male | 1,460 (49.6) | 1,856 (43.7) | < 0.001 |
| Female | 1,484 (50.4) | 2,392 (56.3) |  |
| Age(years), mean ± SD | 81.98 ± 8.96 | 88.45 ± 11.12 | < 0.001 |
| Place of residence, n (%) |  |  |  |
| Urban | 1,265 (43.0) | 1,947 (45.8) | 0.002 |
| Rural | 1,679 (57.0) | 2,301 (54.2) |  |
| Residential status, n (%) |  |  |  |
| Live with families | 2,265 (76.9) | 3,414 (80.4) | < 0.001 |
| Live alone or in a nursing institution | 679 (23.1) | 834 (19.6) |  |
| Smoking habits, n (%) |  |  |  |
| Yes | 533 (18.1) | 642 (15.1) | < 0.001 |
| No | 2,411 (81.9) | 3,606 (84.9) |  |
| Drinking habits, n (%) |  |  |  |
| Yes | 519 (17.6) | 552 (13.0) | < 0.001 |
| No | 2,425 (82.4) | 3,696 (87.0) |  |
| Chronic disease, n (%) |  |  |  |
| Yes | 609 (20.7) | 951 (22.4) | < 0.001 |
| No | 2,335 (79.3) | 3,297 (77.6) |  |
| Social participation, mean ± SD | 12.46 ± 6.46 | 8.53 ± 7.00 | < 0.001 |
| IADL, mean ± SD | 10.77 ± 4.33 | 15.08 ± 6.48 | < 0.001 |

**Notes:** Pearson chi-square tests for categorical variables; One-way analysis of variance tests for continuous variables; IADL, instrumental activities of daily living.

Supplemental Table 2. The fit indices of the all profile models at T1 and T2 based on sensitivity (n = 3,375).

| Time | Profile | AIC | BIC | aBIC | Entropy | VLMR (P) | BLRT (P) | Minimum sizes |
| --- | --- | --- | --- | --- | --- | --- | --- | --- |
| T1 |  |  |  |  |  |  |  |  |
|  | 2 | 34,954.180 | 35,107.280 | 35,027.850 | 0.990 | < 0.001 | < 0.001 | 0.14 |
|  | 3 | 28,996.524 | 29,204.745 | 29,096.712 | 0.974 | 0.043 | < 0.001 | 0.07 |
|  | 4 | 24,947.072 | 25,210.411 | 25,073.780 | 0.982 | 0.003 | < 0.001 | 0.06 |
|  | 5 | 22,340.456 | 22,658.911 | 22,493.683 | 0.985 | 0.062 | 0.038 | 0.02 |
| T2 |  |  |  |  |  |  |  |  |
|  | 2 | 44,872.064 | 45,025.168 | 44,945.731 | 0.983 | 0.001 | < 0.001 | 0.27 |
|  | 3 | 38,181.649 | 38,389.870 | 33,978.039 | 0.969 | 0.004 | 0.002 | 0.22 |
|  | 4 | 34,218.921 | 34,482.259 | 34,345.628 | 0.978 | 0.001 | 0.001 | 0.14 |
|  | 5 | 32,149.608 | 32,468.064 | 32,302.836 | 0.978 | 0.062 | 0.003 | 0.04 |

Abbreviations: AIC, the Akaike information criterion; BIC, the Bayesian information criterion (BIC); aBIC, the Sample-adjusted Bayesian Information Criterion (aBIC); VLMR, the Vuong-Lo-Mendell-Rubin; BLRT, Bootstrapped Likelihood Ratio Test (BLRT).

Supplemental Table 3. Odds Ratio reflecting effects of predictors of different profiles at baseline based on sensitivity analysis (n = 3,375).

| Predictor | Mildly impaired | Moderately impaired | Highly impaired |
| --- | --- | --- | --- |
| Age | 1.09^**^ | 1.12^**^ | 1.12^**^ |
| Cognitive activity | 0.87^**^ | 0.82^**^ | 0.96 |
| Physical activity | 0.92^**^ | 0.55^**^ | 0.73^**^ |
| Social activity | 0.79^**^ | 0.84 | 0.90 |
| Place of residence (ref = rural) | 1.10 | 1.63^*^ | 0.87 |
| Gender (ref = female) | 0.37^**^ | 0.31^**^ | 0.62^*^ |
| Residential status (ref = live alone or in a nursing institution) | 0.77^*^ | 1.23 | 1.45 |
| Smoking habits (ref = no) | 1.22 | 0.89 | 0.86 |
| Drinking habits (ref = no) | 0.75 | 0.80 | 0.52^*^ |
| Chronic disease (ref = yes) | 0.70^**^ | 0.38^**^ | 0.54^**^ |

Ref = Normal function profile.

^*^*P* < 0.05; ^**^*P* < 0.01

Supplemental Table 4. Odds Ratio for covariates predicting transitions among profiles based on sensitivity analysis (n = 3,375).

|  |  | T2 latent profile | | | |
| --- | --- | --- | --- | --- | --- |
| Predictor | T1 latent profile | Normal function | Mildly impaired | Moderately impaired | Highly impaired |
| Age | Normal function | ref | 1.08** | 1.12** | 1.13** |
|  | Mildly impaired | 0.96 | ref | 1.09** | 1.14** |
|  | Moderately impaired | 0.98* | 1.02 | ref | 1.02 |
|  | Highly impaired | 0.96 | 0.90** | 0.98 | ref |
| Cognitive activity | Normal function | ref | 0.94** | 0.96 | 0.92 |
|  | Mildly impaired | 0.98 | ref | 0.97 | 1.05 |
|  | Moderately impaired | 1.10 | 0.91 | ref | 1.18 |
|  | Highly impaired | 1.08 | 0.95 | 1.03 | ref |
| Physical activity | Normal function | ref | 0.99 | 1.00 | 0.95** |
|  | Mildly impaired | 1.06 | ref | 1.04 | 1.02 |
|  | Moderately impaired | 1.13 | 0.94 | ref | 0.82* |
|  | Highly impaired | 1.06 | 1.15** | 1.07** | ref |
| Social activity | Normal function | ref | 0.86* | 0.82 | 0.92 |
|  | Mildly impaired | 1.23 | ref | 1.02 | 1.03 |
|  | Moderately impaired | 0.43 | 1.69 | ref | 0.82 |
|  | Highly impaired | 2.12 | 1.54 | 1.20 | ref |
| Place of residence | Normal function | ref | 0.99 | 0.98 | 1.80* |
| (ref = rural) | Mildly impaired | 0.52* | ref | 0.71 | 1.26 |
|  | Moderately impaired | 2.05 | 3.78 | ref | 2.48* |
|  | Highly impaired | 2.34 | 0.49 | 0.61 | ref |
| Gender | Normal function | ref | 0.66** | 0.68* | 0.68* |
| (ref = female) | Mildly impaired | 1.25 | ref | 0.56 | 0.68 |
|  | Moderately impaired | 1.10 | 1.35 | ref | 0.55 |
|  | Highly impaired | 1.23 | 2.02 | 2.32 | ref |
| Residential status | Normal function | ref | 0.84 | 0.90 | 1.29 |
| (ref = live alone or in a nursing institution) | Mildly impaired | 0.84 | ref | 1.56 | 1.32 |
|  | Moderately impaired | 1.50 | 1.44 | ref | 1.59 |
|  | Highly impaired | 1.35 | 0.24** | 0.49 | ref |
| Smoking habits | Normal function | ref | 0.92 | 0.77 | 0.95 |
| (ref = no) | Mildly impaired | 0.79 | ref | 0.64 | 0.42 |
|  | Moderately impaired | 0.20 | < 0.01a | ref | 0.70 |
|  | Highly impaired | 2.08 | 1.23 | 0.74 | ref |
| Drinking habits | Normal function | ref | 1.21 | 0.94 | 0.79 |
| (ref = no) | Mildly impaired | 0.68 | ref | 1.43 | 1.05 |
|  | Moderately impaired | 5.21 | < 0.01a | ref | 4.13 |
|  | Highly impaired | 3.58 | 2.37 | 1.46 | ref |
| Chronic disease | Normal function | ref | 0.99 | 0.75 | 0.73 |
| (ref = yes) | Mildly impaired | 0.56 | ref | 0.44 | 0.38 |
|  | Moderately impaired | 0.82 | 1.50 | ref | 4.13 |
|  | Highly impaired | 0.75 | 0.44 | 0.66 | ref |

^*^*P* < 0.05; ^**^*P* < 0.01

^a^ Odds Ratio < 0.01.

1. Latent profile analysis at T1

1. Latent profile analysis at T2

Supplemental Fig. 1 The IADL item means for each profile in the 4-profile model at T1 and T2 based on sensitivity analysis (n = 3,375).

I1, visiting neighbors; I2, shopping; I3, making food; I4, washing clothes; I5, walking one kilometer; I6, carrying five kg of weight; I7, crouching and standing three times; I8, taking public transportation.

Supplemental Fig. 2 The percentage in each of the four profiles and transition probabilities from T1 to T2 based on sensitivity analysis (n = 3,375).
